# Supplementary material for: Forecasting temperature and rainfall using deep learning for the challenging climates of Northern India
Source: PeerJ Comput Sci. 2025 Aug 22;11:e3012. doi: 10.7717/peerj-cs.3012 (PMC12453782; doi:10.7717/peerj-cs.3012)
Supplement: Supplemental Information 1 [file peerj-cs-11-3012-s001.docx]

Supplementary Algorithm S1 **Working of Recurrent Neural Network (RNN)**

| **Algorithm 1: Working of Recurrent Neural Network (RNN)** |  |
| --- | --- |
| **Input:** A sequence of data points *x*, denoted as $x=\left[ {x_{t-1,}x}_{t}, x_{t+1}\ldots\right]$ where $x_{t}$represents the input at a specific time step "*t*" within the sequence.   1. The hidden state at time step "*t*", denoted as $f_{t}$, is calculated by combining the current input $x_{t}$with the previous hidden state $f_{t-1}$. This computation is expressed as:   $f_{t}=\sigma\left( \alpha x_{t}+\theta f_{t-1} \right)$  where $\sigma$ represents activation function applied to the sum of weighted inputs, α represents the weight matrix for the connections from the input to the hidden state and θ represents the weight matrix for the connections within the hidden state.   1. The output $y_{t}$ at time step *"t"* can be computed using the hidden state:   $y_{t}=\sigma(\beta f_{t})$  where $\beta$ is the weight matrix for the hidden-to-output connections.  **Output:**  Output at time step *“t”* i.e $y_{t}$ | |
