# Supplementary material for: Forecasting temperature and rainfall using deep learning for the challenging climates of Northern India
Source: PeerJ Comput Sci. 2025 Aug 22;11:e3012. doi: 10.7717/peerj-cs.3012 (PMC12453782; doi:10.7717/peerj-cs.3012)
Supplement: Supplemental Information 2 [file peerj-cs-11-3012-s002.docx]

Supplementary Algorithm S2 **Working of Back Propagation through time (BPPT)**

| **Algorithm 2: Working of Back Propagation through time (BPPT)** |
| --- |
| **Input:** A sequence of data points *x*, denoted as $x=\left[ {x_{t-1,}x}_{t}, x_{t+1}\ldots\right]$ where $x_{t}$represents the input at a specific time step "*t*" within the sequence.   1. The hidden state at time step "*t*", denoted as $f_{t}$, is calculated by combining the current input $x_{t}$with the previous hidden state $f_{t-1}$. This computation is expressed as $f_{t}=\sigma\left( \alpha x_{t}+\theta f_{t-1} \right)$  where $\sigma$, α and θ have the usual meanings.   1. The loss function, such as Mean Squared Error (MSE) or cross-entropy loss, is applied to calculate the loss at each time step. We can represent the loss at a specific time step *‘t’* as $l_{t}$.   The gradients of the loss with respect to the parameters of the network is calculated from the final time step. Subsequently, these gradients are propagated in a backward direction across previous time steps. The gradient at time step *‘t’*, which signifies the impact of the loss on the hidden state at *‘t’* can be represented as: $\frac{\partial l_{t}}{\partial f_{t}}$  1. The gradients are accumulated at each step as they propagate through time ensuring that dependencies between different time steps are captured effectively. Accumulated gradients are represented as $\frac{\partial l}{\partial f_{t}}$ 2. The parameters of the network are updated by utilizing optimization algorithms such as Adam, SGD and RMSprop. The parameter update for each weight can be computed as: $\Delta w=-\eta*\sum\frac{\partial l}{\partial w}$  where $\Delta w$ is the update in weight and $\eta$is the learning rate. |
