# Supplementary material for: Forecasting temperature and rainfall using deep learning for the challenging climates of Northern India
Source: PeerJ Comput Sci. 2025 Aug 22;11:e3012. doi: 10.7717/peerj-cs.3012 (PMC12453782; doi:10.7717/peerj-cs.3012)
Supplement: Supplemental Information 3 [file peerj-cs-11-3012-s003.docx]

Supplementary Algorithm S3 **Working of Long Short Term-Memory**

| **Algorithm 3: Working of Long Short Term-Memory** |
| --- |
| **Input:** A sequence of data points *x*, denoted as $x=\left[ {x_{t-1,}x}_{t}, x_{t+1}\ldots\right]$ where $x_{t}$represents the input at a specific time step "*t*" within the sequence.   1. The forget gate determines whether the information from the previous timestamp should be retained or discarded, based on its relevance. Mathematically, forget gate can be represented as: $F_{t}=\sigma(x_{t}*U_{F}+f_{t-1}*\theta_{F})$  where $\sigma,x_{t},f_{t-1},\theta_{F}$ have the usual meanings and $U_{F}$ is the weight associated with the input. This $F_{t}$ is later multiplied with the cell state of the previous timestamp to determine whether the information is to be kept or not: $S_{t-1}*F_{t}=0 if F_{t}=0$ $S_{t-1}*F_{t}=C_{t-1} if F_{t}=1$  1. The input gate enables the cell to learn and incorporate new information from the current input. $I_{t}=\sigma\left( x_{t}*U_{t}+f_{t-1}*w_{t} \right)$ 2. The updated information to be incorporated into the cell state is determined by the hidden state at the previous timestamp and the input at the current timestamp. $n_{t}=tanh(x_{t}*U_{S}+f_{t-1}*w_{c})$  The activation function used here is $tanh$, which limits the value of the new information between -1 and 1. The cell state is updated as given below: $S_{t}=F_{t}*S_{t-1}+I_{t}*n_{t}$  1. Ultimately, the cell transfers the updated information from the current timestamp to the subsequent timestamp using the output gate, mathematically represented as: $O_{t}=\sigma(x_{t}*U_{O}+f_{t-1}*w_{O})$ 2. The current hidden state is computed as: $f_{t}=O_{t}*tanh(S_{t})$ |
